# Root Cause Analysis of Delayed Emergency Department Computed Tomography Scans

**DOI:** 10.5811/westjem.17831

**Published:** 2024-02-09

**Authors:** Arjun Dhanik, Bryan A. Stenson, Robin B. Levenson, Peter S. Antkowiak, Leon D. Sanchez, David T. Chiu

**Affiliations:** * Beth Israel Deaconess Medical Center, Department of Emergency Medicine, Boston, Massachusetts; † Harvard Medical School, Harvard University, Boston, Massachusetts; ‡ Brigham and Women’s Faulkner Hospital, Department of Emergency Medicine, Boston, Massachusetts; ∘ Co-first authors who equally contributed to this work

## Abstract

**Introduction:**

A solution for emergency department (ED) congestion remains elusive. As reliance on imaging grows, computed tomography (CT) turnaround time has been identified as a major bottleneck. In this study we sought to identify factors associated with significantly delayed CT in the ED.

**Methods:**

We performed a retrospective analysis of all CT imaging completed at an urban, tertiary care ED from May 1–July 31, 2021. During that period, 5,685 CTs were performed on 4,344 patients, with a median time from CT order to completion of 108 minutes (Quartile 1 [Q1]: 57 minutes, Quartile 3 [Q3]: 182 minutes, interquartile range [IQR]: 125 minutes). Outliers were defined as studies that took longer than 369 minutes to complete (Q3 + 1.5 × IQR). We systematically reviewed outlier charts to determine factors associated with delay and identified five factors: behaviorally non-compliant or medically unstable patients; intravenous (IV) line issues; contrast allergies; glomerular filtration rate (GFR) concerns; and delays related to imaging protocol (eg, need for IV contrast, request for oral and/or rectal contrast). We calculated confidence intervals (CI) using the modified Wald method. Inter-rater reliability was assessed with a kappa analysis.

**Results:**

We identified a total of 182 outliers (4.2% of total patients). Fifteen (8.2%) cases were excluded for CT time-stamp inconsistencies. Of the 167 outliers analyzed, 38 delays (22.8%, 95% confidence interval [CI] 17.0–29.7) were due to behaviorally non-compliant or medically unstable patients; 30 (18.0%, 95% CI 12.8–24.5) were due to IV issues; 24 (14.4%, 95% CI 9.8–20.6) were due to contrast allergies; 21 (12.6%, 95% CI 8.3–18.5) were due to GFR concerns; and 20 (12.0%, 95% CI 7.8–17.9) were related to imaging study protocols. The cause of the delay was unknown in 55 cases (32.9%, 95% CI 26.3–40.4).

**Conclusion:**

Our review identified both modifiable and non-modifiable factors associated with significantly delayed CT in the ED. Patient factors such as behavior, allergies, and medical acuity cannot be controlled. However, institutional policies regarding difficult IV access, contrast administration in low GFR settings, and study protocols may be modified, capturing up to 42.6% of outliers.

## INTRODUCTION

A solution for emergency department (ED) congestion remains elusive. As reliance on imaging grows, computed tomography (CT) turnaround time—from CT order to completion—has been identified as a major bottleneck.^1^ ^,^ ^2^ One study showed that patients who had radiological diagnostics were 4.4 times more likely to stay over four hours in the ED than those who did not have these tests.^2^ Numerous studies have identified strategies to decrease CT turnaround times. White et al mapped the complex process of ED radiology transport and applied systems engineering principles to improve efficiency without increasing resource use.^3^ Perotte et al assembled a multidisciplinary stakeholder team to identify barriers and implement solutions to reduce CT turnaround time from 5.8 to 4.6 hours despite a 13.8% increase in the number of scans.^1^ Various studies have demonstrated the efficacy of applying Lean and Six Sigma principles.^4^ ^,^ ^5^ Finally, queuing theory has been used to model ED delays with varying levels of resource utilization.^6^ ^,^ ^7^

There has not yet been a dedicated outlier analysis of delayed CT scans in the ED. In this study we sought to identify factors associated with significantly delayed CT. This is consequential given that patients with ED stays longer than six hours directly contribute to crowding.^8^

## METHODS

We performed a retrospective analysis of all CTs completed at an urban, tertiary care ED in Boston, Massachusetts, from May 1–July 31, 2021. During that period, 5,685 scans were performed on 4,344 patients, with a median time from CT order to completion of 108 minutes (Quartile 1 [Q1]: 57 minutes, Quartile 3 [Q3]: 182 minutes, interquartile range [IQR]: 125 minutes). Outliers were defined as studies that took longer than 369 minutes to complete (Q3 + 1.5 × IQR). We defined CT completion time as the point at which the CT technologist marks the study as completed, thereby removing the confounder of radiologist read time.

We systematically reviewed outlier charts and communications between members of the care team to determine factors associated with delay and identified five factors: behaviorally non-compliant or medically unstable patients; intravenous (IV) line issues (eg, IV infiltration, difficult IV access, inadequate IV size); contrast allergies; glomerular filtration rate (GFR) concerns; and delays related to imaging protocol (eg, need/request for contrast administration, including IV, oral, and/or rectal). Confidence intervals (CI) were calculated using the modified Wald method. We performed a kappa analysis to assess for inter-rater reliability. This was done on each category individually as some outlier cases had multiple contributing factors. This study design was approved by our institutional review board with a determination of exemption. We observed 10 of the 12 methods of health record review as outlined by Worster et al, with the exceptions of abstractor performance monitoring and abstractor blinding to hypothesis.^9^

## RESULTS

We identified 182 outliers (4.2% of total patients) and excluded 15 cases (8.2%) for CT time-stamp inconsistencies. Of the 167 outliers analyzed, 38 delays (22.8%, 95% CI 17.0–29.7) were due to behaviorally non-compliant or medically unstable patients; 30 (18.0%, 95% CI 12.8–24.5) were due to IV issue;, 24 (14.4%, 95% CI 9.8–20.6) were due to contrast allergies; 21 (12.6%, 95% CI 8.3–18.5) were due to GFR concerns; and 20 (12.0%, 95% CI 7.8–17.9) were related to imaging study protocol. The cause of the delay was unknown in 55 cases (32.9%, 95% CI 26.3–40.4). The distribution of CT types for outlier cases is illustrated in Table 1.

**Table 1.** Distribution of outliers in emergency department computed tomography.

Computed tomography type	Number (% total)
Torso (any chest/abdomen/pelvis imaging)	124 (62.0%)
Non-contrast head	37 (18.4%)
Spine	15 (7.5%)
Angiogram head and neck	13 (6.5%)
Face, orbits, soft tissue neck	7 (3.5%)
Extremity	5 (2.5%)

Kappa values ranged from 0.69–0.98 for all the categories (Table 2). Intravenous issues had the lowest degree of agreement, while delays due to allergy protocols had the highest degree of agreement.

**Table 2.** Kappa analysis of factors associated with significantly delayed computed tomography.

Factors associated with delay	Kappa (95% confidence interval)
Intravenous line issues	0.69 (0.55–0.83)
Contrast allergy	0.98 (0.93–1.00)
Renal function concerns	0.86 (0.74–0.98)
Behaviorally or medically unstable patient	0.85 (0.75–0.94)
Imaging protocol	0.83 (0.70–0.96)
Unknown	0.86 (0.78–0.95)

## DISCUSSION

Our review identified both modifiable and non-modifiable factors associated with significantly delayed CT in the ED. Patient factors such as behavior, allergies, and medical acuity cannot be controlled. However, institutional protocols regarding difficult IV access, contrast administration in low GFR settings, and study protocols may be modified. One of these modifiable factors is IV access: early involvement of an IV team or utilization of ultrasound for IV placement may expedite imaging. Our data suggests that 18.0% of outliers can be more efficiently imaged by improving IV placement strategies. Studies have shown that nearly 9% of ED patients have difficult IV access, defined in one paper as requiring ≥3 attempts or an ultrasound-guided line. These patients experience statistically significant delays in establishing IV access, obtaining lab results, and receiving analgesia, as well as experiencing longer ED length of stay.^10^ Therefore, the benefits of expeditious IV placement extends beyond enhanced CT throughput.

The second modifiable factor pertains to contrast administration in low GFR settings. There is growing evidence that the risk of acute kidney injury resulting from contrast administration in patients with reduced GFR may have been overestimated.^11^ This shift has been attributed to the fact that much of the existing literature was not sufficiently controlled to distinguish between contrast-induced and contrast-associated nephropathy.^11^ Institutions may consider revising policies, such as forgoing mandatory pre-hydration or radiologist conversations and amending exiting GFR cutoffs, to expedite imaging.

Judicious protocoling of CT may address a proportion of outliers. One study found that patients who had an abdominal/pelvic CT with only IV contrast had an approximately two-hour shorter ED length of stay when compared to patients who received a CT with oral and IV contrast.^12^ This difference was even more pronounced when comparing patients who underwent CT with oral contrast with those who were imaged with no contrast: patients who received no contrast had an approximately four-hour shorter length of stay.^13^ Finally, elimination of the routine use of oral contrast in abdominal/pelvic CT has been shown to shorten ED length of stay without affecting diagnostic accuracy.^14^ Considered use of contrast may improve CT throughput.

We modified ED workflow to improve CT throughput and address some of the outliers identified in this study. We revised institutional policies regarding contrast administration in low GFR patients and streamlined communication between the ED and radiology teams. Previously, CT in a patient with a GFR of 45–60 milliliters per minute (mL/min) triggered a conversation between radiology and the ED care team regarding oral hydration. Under the new guidelines, patients with a GFR ≥45 mL/min may proceed directly to CT with IV contrast. For GFR 30–45 mL/min, radiology will call the ED team and discuss the merits of administering IV contrast. If the ED team elects to proceed with IV contrast, the volume, timing (pre- or post-CT), and route of fluid hydration are all at the discretion of the ED. Computed tomography throughput is therefore maximized as patients may be hydrated *after* receiving CT. For cases with a GFR ≤30 mL/min, radiology will discuss the merits of IV contrast with the ED team. If contrast is to be administered, one hour of IV pre-hydration is recommended prior to imaging if there is no contraindication. Communication between the ED and radiology teams has been streamlined with the introduction of automated messages that indicate when pre-hydration has been initiated and completed.

Analysis of the communication between the radiology and ED teams revealed that there were often multiple calls regarding a patient’s hydration status. We intend to repeat a similar analysis with the above interventions to assess for a change in the number of delayed CT studies due to GFR concerns. We recommend that institutions perform their own analysis of outliers to understand opportunities for improvement and to expedite overall ED throughput.

## LIMITATIONS

Limitations of our study include the fact that it was conducted at a single, urban, academic, tertiary-care ED. This population may not be indicative of that seen by other EDs. Furthermore, residents in our ED take ownership of difficult IV placement as part of their training. Thus, difficult IV placement may not be associated with delayed CT in other EDs that have dedicated IV access teams. The GFR cutoffs for contrast administration in our ED are admittedly stringent. Other institutions with less stringent cutoffs may not see as many significantly delayed CT studies due to GFR concerns.

For the purposes of this analysis, patient factors such as behavior, allergies, and medical acuity were considered non-modifiable. Future studies may consider reviewing protocols for allergy prophylaxis or behavioral de-escalation. Finally, we excluded a total of 41.1% of outliers: 8.2% due to CT time-stamp inconsistencies and 32.9% because the cause of the delay could not be identified despite thorough review of outlier charts.

## CONCLUSION

We identified modifiable and non-modifiable factors associated with significantly delayed CT in the ED. Interventions such as prompt IV team involvement or utilization of ultrasound for IV placement, revision of institutional policies regarding contrast administration in low GFR settings, and CT protocol consideration may address up to 42.6% of outliers. These interventions may improve CT turnaround times and ED throughput. Future research will extend this analysis by measuring the effect of revised institutional policies regarding contrast administration.
